# The *vasa *regulatory region mediates germline expression and maternal transmission of proteins in the malaria mosquito *Anopheles gambiae*: a versatile tool for genetic control strategies

**DOI:** 10.1186/1471-2199-10-65

**Published:** 2009-07-02

**Authors:** Philippos A Papathanos, Nikolai Windbichler, Miriam Menichelli, Austin Burt, Andrea Crisanti

**Affiliations:** 1 Imperial College London, Division of Cell and Molecular Biology, Imperial College Road, London SW7 2AZ, UK; 2 Dept of Biology and Centre for Population Biology, Imperial College London, Silwood Park, Ascot, Berks, SL5 7PY, UK

## Abstract

**Background:**

Germline specific promoters are an essential component of potential vector control strategies which function by genetic drive, however suitable promoters are not currently available for the main human malaria vector *Anopheles gambiae*.

**Results:**

We have identified the *Anopheles gambiae vasa*-like gene and found its expression to be specifically localized to both the male and female gonads in adult mosquitoes. We have functionally characterised using transgenic reporter lines the regulatory regions required for driving transgene expression in a pattern mirroring that of the endogenous *vasa *locus. Two reporter constructs indicate the existence of distinct *vasa *regulatory elements within the 5' untranslated regions responsible not only for the spatial and temporal but also for the sex specific germline expression. *vasa *driven eGFP expression in the ovary of heterozygous mosquitoes resulted in the progressive accumulation of maternal protein and transcript in developing oocytes that were then detectable in all embryos and neonatal larvae.

**Conclusion:**

We have characterized the *vasa *regulatory regions that are not only suited to drive transgenes in the early germline of both sexes but could also be utilized to manipulate the zygotic genome of developing embryos via maternal deposition of active molecules. We have used computational models to show that a homing endonuclease-based gene drive system can function in the presence of maternal deposition and describe a novel non-invasive control strategy based on early *vasa *driven homing endonuclease expression.

## Background

Mosquito species of the *Anopheles gambiae *complex are the major vectors of the human malaria parasite *Plasmodium falciparum *and pose an enormous burden on human health and economies. Every year about 300 million people are infected by *Plasmodium *parasites and over a million children die as consequence of malaria infection[1]. While many insect pests species have long been successfully targeted by population control measures such as insecticides, for others, including *A. gambiae*, many countries lack the resources and the logistics to successfully implement these measures over prolonged periods of time. Alternative vector control strategies that are affordable, easy to implement and sustainable are needed. The search for new solutions has prompted an unprecedented research effort aimed at generating new molecular tools and a better understanding of the biology and genetics of *Anopheline *mosquitoes that resulted in the sequencing of the *A. gambiae *genome [2] and the development of gene transfer technology for a series of vector species [3-5]. These advances have made it possible to generate genetically manipulated mosquitoes expressing genes that block the transmission of malaria in experimental systems [6-9]. The translation of any such achievements into suitable control measures would require the development of strategies to spread the genetic modification from few laboratory reared insects to large wild type populations, since population replacement strategies based on the massive release of genetically modified mosquitoes carrying a desired trait such as malaria refractoriness are obviously difficult to implement. This represents a major scientific and technical challenge. Recently genetic drive systems, based on naturally occurring 'selfish' genetic elements have been proposed to overcome some of these challenges. A number of candidates have been proposed including transposable elements, MEDEA complexes or homing endonuclease genes (HEGs) [10-13]. HEGs are highly specific DNA endonucleases that promote the movement of their encoding DNA from one allele to the other by creating a double-strand break (DSB) at a specific, long (15–40 bp) target site in the allele that lacks the HEG [14,15]. The host cells DSB repair machinery then uses the homologous chromosome containing the HEG containing allele as a template for repair, converting what was initially a wild type allele into one that contains the HEG, a process called homing. The observation that HEGs can be engineered to cleave novel DNA sequences [16-19] offers a multitude of opportunities to utilize these elements for mosquito control. For example, HEGs could be used to drive genes that confer parasite refractoriness through a mosquito population. Alternatively, HEGs designed to target an essential mosquito gene or a gene required for female fertility could be utilized to introduce a genetic load on the population leading to population size reduction or collapse [12]. For homing to lead to genetic drive and thus have an effect in the spread of HEGs in mosquito populations, homing would have to occur in cells of the germline and only expression of the endonuclease in germline cells prior to meiosis would ensure the availability of the homologous chromosome, and thus the HEG allele as a repair template, prior to chromosomal disjunction. Expression of a transgene in this pattern requires the availability of suitable regulatory sequences. The regulatory regions of the *β2-tubulin *gene have been utilized to drive transgene expression during late, predominantly postmeiotic stages of spermatogenesis in *Anopheles stephensi *and *gambiae *[20,21]. The female germline-specific regulatory elements of the nanos gene have been used to express transgenes in the mosquito vector species *Aedes aegypti *[22]. However, a regulatory sequence that would allow sufficiently early pre-meiotic expression in both male and female germlines suitable for a HEG strategy is not currently available for *Anopheles*. One of the first genes to be expressed in the germline of *Drosophila *and some other species is *vasa*, which encodes a protein of the DEAD-Box RNA helicase family [23]. *vasa *is essential for embryonic patterning, the assembly of the pole plasm and germ cell function [24-27]. The pole plasm serves essential functions for the formation of the embryonic pole cells, the progenitor cells of the *Drosophila *germline. Pole cells form at the posterior pole of the syncytial blastoderm embryo and then migrate into the abdominal region of the embryo, where they are encapsulated by somatic gonadal mesodermal cells to form the embryonic gonads [28]. In *Drosophila*, zygotic germline vasa expression starts immediately after gastrulation and continues into oogenesis and spermatogenesis persisting throughout the adult life cycle [26,27,29]. The regulatory regions of this gene have been used successfully to drive expression of ransgenes in the *Drosophila *germline [30,31] and could therefore represent an excellent candidate for an early germline-specific regulatory element for *Anopheles *mosquitoes. Here we report the identification and the expression pattern of the *A. gambiae vasa *orthologue using transgenic reporter strains. We show that the activity of this *Anopheles *regulatory region depends on distinct elements and that such activity is suitable not only for the expression of transgenes in both the male and the female germline prior to meiosis, but also allows us to propose a novel inundative genetic control strategy that combines the advantages of genetic drive and dominant sterility.

## Results

### Identification and characterization of the *Anopheles gambiae vasa *orthologue

**Figure 1:**
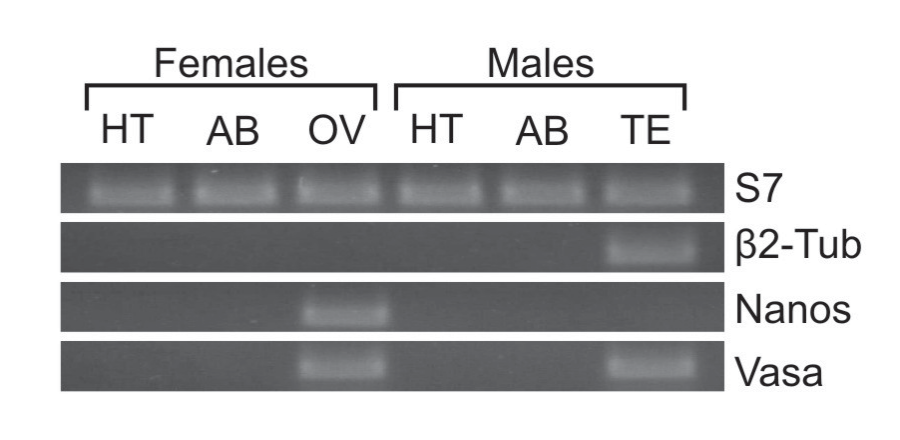
**Expression analysis of the *A. gambiae vasa *orthologue AGAP008578**. Tissue specific expression analysis using RT-PCR. We used cDNA from dissected adult male and female tissues including the head and thorax (HT), abdomen (AB) ovaries (OV) and testis (TE) to amplify mRNAs of the *vasa *gene and as control the nanos, beta2-tubulin genes and the S7 genes.

**Figure 2:**
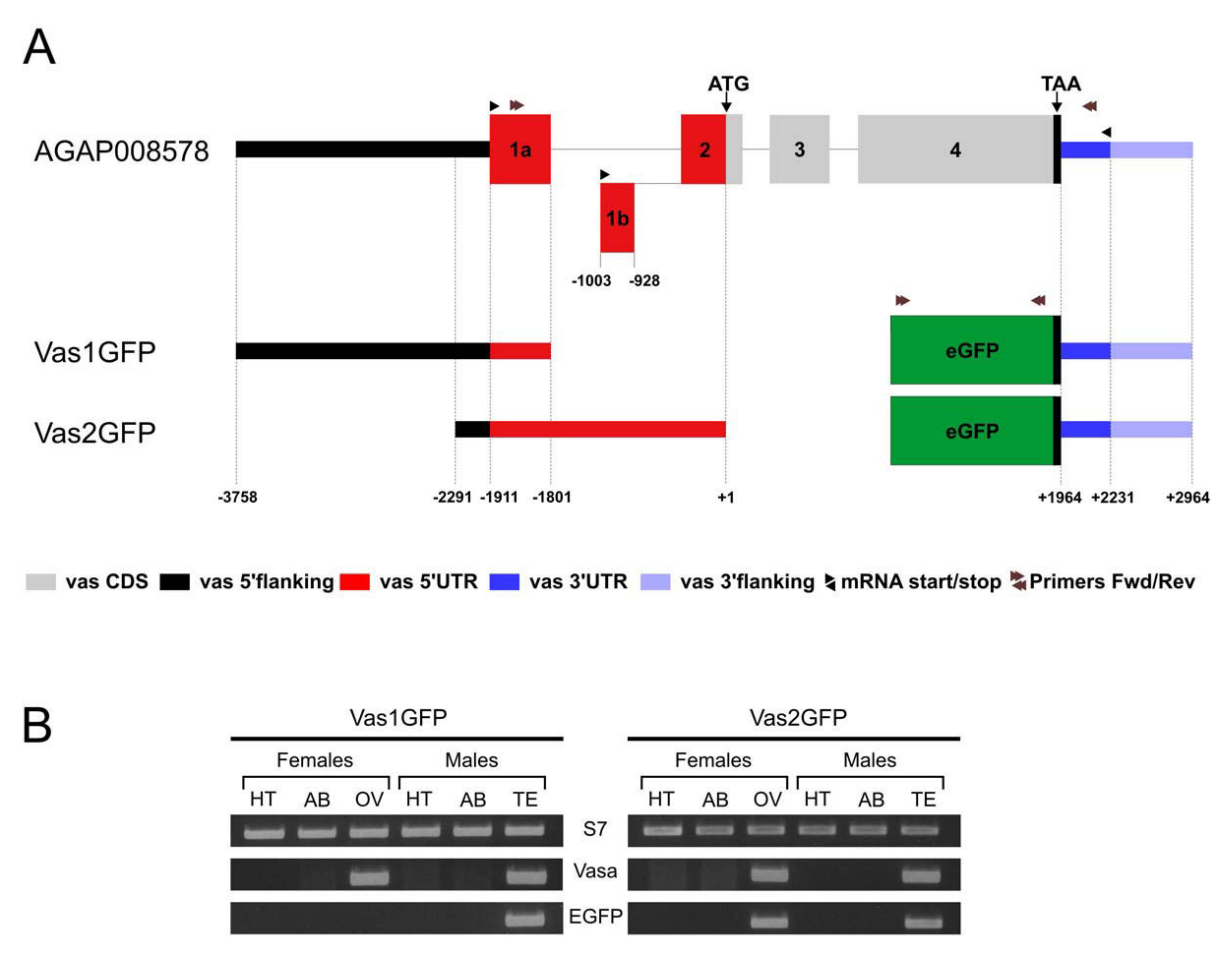
**Transgenic constructs and expression pattern of eGFP in transgenic adult mosquitoes**. (A) Schematic diagram of the *Anopheles gambiae vasa *locus and the Vas1-GFP and Vas2-GFP transgenic constructs. (B) RT-PCR expression analysis of transgenic lines. We used cDNA from transgenic adult Vas1GFP and Vas2GFP mosquitoes of head and thorax (HT), abdomen (AB) ovaries (OV) and testis (TE) to determine expression of endogenous *vasa*, eGFP and the S7 control gene.

Using the *D. melanogaster *Vasa as a template, AGAP008578 was identified as the likely *A. gambiae *orthologue with an overall amino acid sequence identity of 49%. A likely orthology relationship with vasa was confirmed by a reverse blast of AGAP008578 against the *D. melanogaster *genome. High level sequence conservation was observed in putative RNA and ATP interacting residues. Regions spanning canonical DEAD-box RNA helicase family domains, including all motifs within the two tandemly repeated RecA-like domains, were the most conserved (see Additional File 1). N-terminal sequences, which are unique to *vasa *and *Dep1p *orthologues showed lower levels of conservation. The RNA-interacting residues arginine 403 and glutamine 497 that distinguish Vasa from other *Drosophila *DEAD box helicases [32] were also present in AGAP008578 within their conserved motifs. To establish the expression profile of AGAP008578 we performed reverse transcriptase-PCR (RTPCR) on total mRNA extracted from dissected adult tissues of wild type *A. gambiae *(G3 strain). The analysis demonstrated that AGAP008578 transcripts could only be found in testes and ovaries. All somatic tissues tested, including the head, thorax and abdomen from male and female mosquitoes, did not show detectable levels of transcript (Figure 1). We therefore concluded that AGAP008578 is the *A. gambiae vasa*-like gene; for simplicity this gene will be referred to as *vasa *for the remainder of this report. We established the organisation of the *Anopheles vasa *locus by combining available *A. gambiae *EST clusters (AnoEST), rapid amplification of cDNA ends (RACE) experiments (J. Meredith personal communication) and in-silico exon prediction. Together these data indicated that two alternative *vasa *transcripts are generated by the alternative utilization of either one of the first two exons both mapping in the 5' untranslated region (Figure 2A). Both types of transcript were found in all tissues of both sexes that show *vasa *expression (data not shown) and the significance of these alternative transcripts is unknown.

### Generation of transformation constructs Vas1GFP and Vas2GFP

Based on our understanding of the organisation of the *vasa *locus we generated two reporter constructs pBac{3XP3-RFP}Vas1-eGFP and pBac{3XP3-RFP}Vas2-eGFP. Vas1GFP and Vas2GFP reporters were designed to drive expression of eGFP from putative regulatory regions of the *A. gambiae vasa *gene (AGAP008578). Approximately 2 kb putative regulatory sequence (-3758 to -1911) upstream of the first predicted transcription start site was included in Vas1GFP together with exon 1a (-1911 to -1801), In an attempt to minimize the size of the Vas1GFP expression cassette all subsequent sequences downstream of exon 1a of the 5'UTR leading up to the start codon were omitted. In contrast, Vas2GFP was designed to contain a much shorter regulatory sequence (0.3 kb) upstream of the transcription initiation site (-2291 to -1911) but included the entire *vasa *5'UTR starting from exon 1a (-2291 to +1) and leading up to the start codon (Figure 2A). At the 5' end the two constructs therefore shared only 380 base pairs upstream of the first transcription start site and exon 1a. For both constructs a sequence of 1 kb starting from the *vasa *stop codon (+1964 to +2965) containing the entire *vasa *3' UTR was utilized 3' of the eGFP stop codon. In addition both constructs contained the 3xP3-RFP cassette as a visual marker and piggyBac inverted repeats for transposasemediated integration.

### The Vas1GFP regulatory regions drive expression in male spermatogenesis

**Figure 3:**
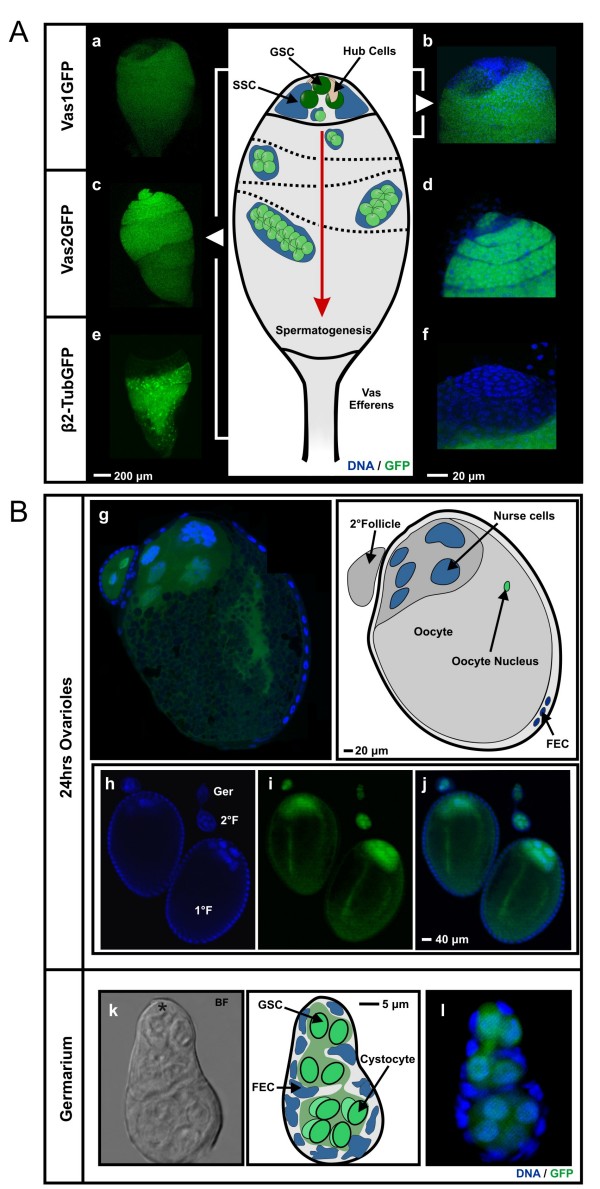
**Confocal analysis of eGFP expression in transgenic gonads**. (A) The middle panel depicts the organisation of an *Anopheles gambiae *testis. In the apical tip the stem cell niche is sustained by a set of somatic cells, called hub cells (pink), which regulate the maintenance of the GSC (dark green) and SSC (dark blue) populations. Upon replication of the GSC, one of the two resulting daughter cells will differentiate into a primary spermatogonium (light green) and begin the developmental maturation process to become mature sperm. Testicular expression of eGFP from transgenic males of Vas1GFP (panels a, b), Vas2GFP (panels c, d) and β2-Tubulin-eGFP (panels e, f). Distribution of eGFP in the entire testes (panels a, c, e). Micrographs of the apical hub regions of transgenic testes (panels b, d, f). The eGFP expression in the GSC region is limited to Vas2GFP (panel d) and is absent in both Vas1GFP (panel b) and in β2-Tubulin-eGFP (panel f). (B) Expression of eGFP in ovarian follicles (panel g, h, i, j) and in germaria (panels k brightfield; l fluorescence). eGFP is expressed in nurse cells of all developing follicles and transported to the oocyte cytoplasm (panel g, i). In germaria eGFP clearly labels germline stem cells (green, GSCs) and developing cystocytes (light green) (panel l). Expression is not detectable in cells of the follicular epithelium (blue, FEC).

Three independent transgenic *A. gambiae *lines were generated using the Vas1GFP construct. In all three transgenic lines the expression of eGFP was detectable exclusively in the vicinity of the developing gonads. Expression of eGFP was only detectable in approximately half of the transgenic larvae, irrespective of the sex of the transgenic parent. When separated and grown to adulthood all GFP positive larvae emerged as males and all GFP negative larvae as females (see Additional File 2A). To examine this phenotype further, RT-PCRs were performed on dissected adult tissues which revealed that eGFP transcript from Vas1GFP was only detectable in transgenic testes, unlike the endogenous *vasa *transcript which appeared in both testes and ovaries (Figure 2B). Exclusive activity of Vas1GFP in testes was further verified by western-blotting with anti-eGFP antibody (see Additional File 2D), confirming that absence of eGFP fluorescence in ovaries results from the absence of transcription from the Vas1GFP construct. To confirm this result we generated two *Anopheles stephensi *transgenic lines using the Vas1GFP construct (data not shown). Transgenics of this related vector species showed an identical eGFP expression pattern indicating that the Vas1GFP construct lacked the regulatory sequences required for transcription in female ovaries. The tissue specific expression pattern from Vas1GFP was similar to that reported for the *A. gambiae *testis specific promoter, β2-tubulin [21]. We compared eGFP fluorescence in developing larval stages and found that unlike the β2-tubulin promoter, in which testes-specific eGFP staining is only detectable in late 3^rd ^instar larvae (L3), eGFP fluorescence from Vas1GFP was detectable in neonatal larvae (L1). Confocal analysis of dissected testes from Vas1GFP transgenic males revealed a widespread distribution of eGFP signal along the longitudinal axis of the organ (Figure 3A, panel a) ranging from the gonial amplification stages, developing spermatocysts up to individual mature sperm cells. Sperm transversing the vas efferens or removed from WT female spermathaeca showed cytoplasmic localization of eGFP (see Additional File 2B). When compared to testes from transgenic β2-tubulin eGFP reporter lines, the eGFP expression in Vas1GFP males was, albeit weaker overall, starting slightly closer to the hub of the organ (Figure 3A panels b, f), thus indicating that transcription (or translation) from Vas1GFP is likely initiated at an earlier stage of spermatogenesis than β2-tubulin. Vas1GFP expression was however not detectable in the apical tip of the testes indicating that the included regulatory regions did not direct expression in male germline stem cells (GSCs) (Figure 3A panel b).

### Vas2GFP drives eGFP expression during early male spermatogenesis and female oogenesis

We generated three independent transgenic *A. gambiae *lines using the Vas2GFP construct. In these mosquitoes eGFP was detectable in all larvae, regardless of sex, in the vicinity of the developing germline. As in Vas1GFP lines, fluorescence was detectable immediately upon hatching in L1 larvae and persisted throughout development. We were not able to detect any differences in the morphology of the fluorescent germlines between sexes of early larvae (L1–L3). In L4 larvae and pupae, ovaries appeared as rod-like longitudinal structures whereas the testes adopted a more spherical-like shape, allowing robust larval sexing (see Additional File 2C). RT-PCR experiments performed on dissected adult tissues from Vas2GFP transformants revealed that eGFP transcript was detectable in gonads of both sexes but not in somatic tissues, mirroring the expression pattern of the endogenous *vasa *gene (Figure 2B). In dissected testes from Vas2GFP lines, the eGFP distribution pattern was detectable in all stages of spermatogenesis including the GSCs in the apical tip (Figure 3A, panel c and d). DAPI staining of the hub region demonstrated that cells in the anterior tip of the testis were expressing eGFP thus indicating that the regulatory regions of Vas2GFP were active in GSCs. Below the GSCs a ring of cells, presumably the somatic stem cells (SSCs) surrounding the GSCs, did not show any fluorescence, supporting the observation that in testes *vasa *is only expressed in germline progenitor cells and developing spermatocytes and not in supporting somatic cells. To investigate the activity of the *vasa *promoter in developing oocytes, ovaries from Vas2GFP heterozygous females were fixed and counterstained with DAPI for confocal analysis 24 and 48 hours post-blood-feeding. All oocytes examined exhibited similar levels of green fluorescence indicating that eGFP expression was not dependent on the genotype of the oocyte nucleus (see below). Fluorescence was distributed around the seven nurse cells of both primary and secondary follicles in a uniform pattern (Figure 3B, panel g, i). Within the oocyte, a distinct band of eGFP fluorescence advancing from the adjacent nurse cells in an anteroposterior direction towards the posterior pole was detectable (Figure 3B panel g, i). Germaria were also clearly labelled with eGFP, indicating that Vas2GFP is active in female GSCs. (Figure 3B panel k, l). The central mass of the germarium, containing the germline stem cells and cystoblasts [33], showed ubiquitous distribution of eGFP fluorescence, staining both the nuclei and the cytoplasm. eGFP was not detectable in the follicular epithelial cells of the germarium or the ovarian follicle (Figure 3B panels i, j and l). Apart from size we found no obvious difference in the expression pattern of ovarian follicles developing at 24 hour and 48 hour after blood feeding (data not shown). On the basis of these findings we concluded that sequences present in the Vas2GFP construct but absent from Vas1GFP are required for the expression of *vasa *in the female and early male germline cells including the GSCs.

### Maternal deposition of eGFP in Vas2GFP embryos

**Figure 4:**
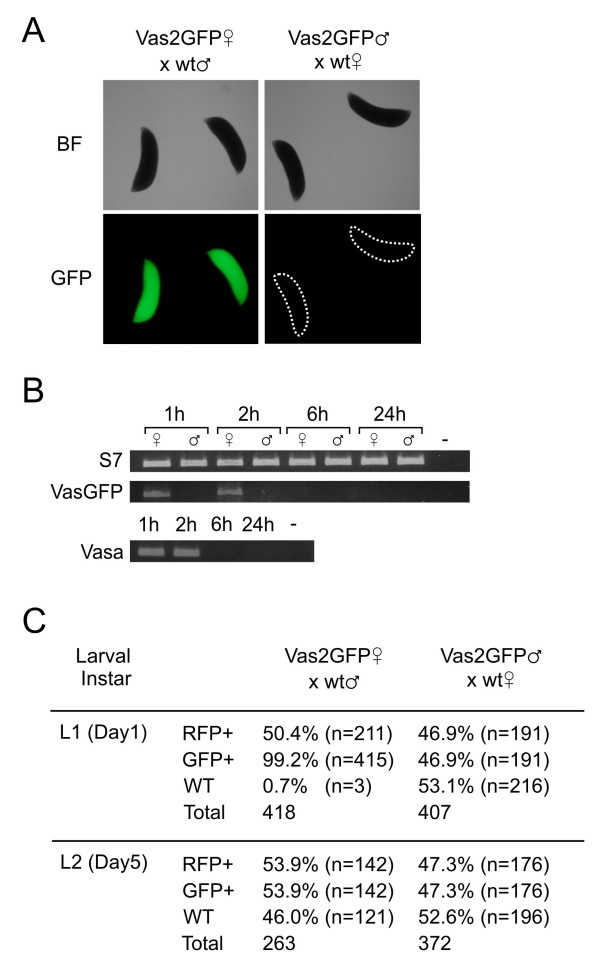
**Maternally derived eGFP deposited in embryos of Vas2GFP transgenic mosquitoes**. (A) Brightfield (upper panels) and fluorescent micrographs (lower panels) of embryos deriving from heterozygous transgenic Vas2GFP mothers (left) or Vas2GFP fathers (right) crossed to wild type. (B) RT-PCR analysis of eGFP transcript deposition in laid embryos deriving from transgenic Vas2GFP mothers or Vas2GFP fathers in a time course from 1 hour post-oviposition until 24 hours post-oviposition. C) Larvae deriving from either heterozygous Vas2GFP mothers crossed to wild type males or heterozygous Vas2GFP fathers crossed to wild type females scored for eGFP (maternal deposition) and RFP fluorescence (segregation of transgene). Scoring was performed in all hatched larvae immediately upon hatching (L1 larval stage) and 5 days later (L2 larval stage).

In *Drosophila, vasa *protein and mRNA are deposited into developing oocytes as maternal derived factors [27,29]. We observed that 72 hours post-blood-feeding, developing oocytes of Vas2GFP transformant female mosquitoes contained ubiquitous distribution of eGFP signal (data not shown). To investigate maternal deposition of the eGFP transgene by our *vasa *regulatory sequence, we mated Vas2GFP transgenic females to wild type males and, as a control, Vas2GFP transgenic males to wild type females. Maternal deposition of eGFP from Vas2GFP heterozygous females was readily detectable in uniformly fluorescent embryos and neonatal hatchlings but not in control embryos or hatchlings, where the transgene was transmitted from transgenic fathers (Figure 4A). The phenotype of nearly all larvae from heterozygous Vas2GFP maternal crosses was eGFP positive (uniformly staining the larvae), whilst the transgene, detected by the 3xP3-RFP marker, segregated to the expected 50% of the progeny. In progeny originating from crosses of Vas2GFP transgenic males to wild type females the eGFP phenotype (restricted to the gonads) was linked to the expression of the RFP marker (Figure 4C). We extracted total mRNA from embryos at several time points after oviposition and performed RT-PCRs to specifically amplify both the endogenous *vasa *and engineered eGFP transcript. Similarly to what was observed for *vasa*, the eGFP transcript could be detected up to 2 hours after oviposition (Figure 4B) thus showing that this promoter directs the transfer of both protein and mRNA from transgenic follicles to offspring of the next generation.

### Impact of maternal deposition on a HEG-based invasive control strategy

**Figure 5:**
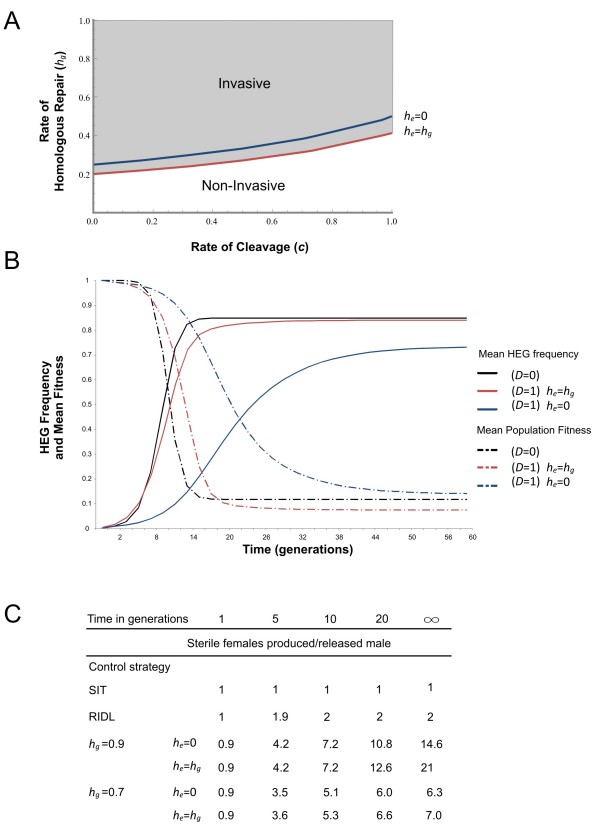
**Population modelling of *vasa*-driven HEGs targeting female-specific genes**. (A) and (B) HEG constructs targeting a somatic female gene for invasive vector control, (C) HEG constructs targeting a germline-specific female gene for non-invasive vector control. A) The threshold rate of homologous repair in the germline (*h*_*g*_) needed for the HEG to invade a population from low frequency, as a function of the overall rate of cleavage. Invasiveness of such a construct is defined as its ability to spread from rare through a population by virtue of genetic drive. The threshold differs depending upon the rate of homing in the embryo (*h*_*e*_) (red vs. blue lines). Conversely, with no maternal deposition, the HEG will invade for any *h*_*g*_>0. (B) Frequency of the HEG and population mean fitness assuming *c *= 0.9, *h*_*g *_= 0.9, and an initial release frequency of 1%. Black line: no maternal deposition (*D *= 0); red line: maternal deposition in which homing rates in the embryo mirror those in the germline (*D *= 1; *h*_*e *_= *h*_*g*_); blue line: maternal deposition in which all cut sites are repaired exclusively by non-homologous repair (*D *= 1; *h*_*e *_= 0). (C) Number of sterile females produced per released male for the HEG-based non-invasive strategy proposed, compared to classical inundative control strategies. To be able to compare to classical measures constructs are released in homozygote males assuming *c *= 0.9 and for simplicity the models assume that on average one male mates once in his lifetime. *c *rate of cleavage); *h *(rate of homologous repair); *D *(maternal deposition).

Based on the properties of the Vas2GFP construct we set out to investigate how the expression characteristics of the *A. gambiae vasa *regulatory region would affect its performance in potential vector control strategies. In particular, we were interested to determine how maternal deposition would affect the effectiveness of HEG-based control methods that aim to reduce mosquito population numbers by imposing a genetic load. Previous models have considered a HEG designed to target a somatically expressed gene essential for female viability or fertility, whose knockout is recessive but has no effect on male fitness [12]. The models predicted that if such a HEG is released at low frequencies into a population it would invade and reach an equilibrium frequency. The maximum equilibrium frequency achievable would depend upon the efficiency of target cleavage and the relative frequencies of homologous repair (HR) and non-homologous repair (NHR) [12,34]. In our model, by virtue of maternal deposition, the homing endonuclease is also active against the zygotic genome of embryos originating from females carrying the HEG allele. Homozygous mutant offspring, inviable or sterile in the case of females, can thus arise by inactivation of the target genes via HR or NHR in embryos even if the paternally derived allele is originally wild-type. Since maternal deposition of the homing endonuclease can reduce the reproductive fitness of heterozygote females, because their daughters are inviable or sterile, the HEG will not necessarily spread from rare, but instead will only invade the population if the rate of repair by homologous recombination is above a threshold value in reference to a particular rate of cleavage (Figure 5A). Moreover, the equilibrium frequency of a HEG that is maternally deposited will significantly depend on the outcome of HEG-induced cleavage in the embryo. HEG equilibrium frequencies remain unaffected, when the relative rate of HR in embryos mirrors that of the germline (*h*_*e *_= *h*_*g*_), but when all cut sites in embryos are repaired by NHR (*h*_*e *_= 0) then the equilibrium frequency is lowered (Figure 5B). The genetic load imposed by a maternally deposited HEG can be higher, due to the extra lethality or sterility. Reduced population fitness can therefore be achieved, although this would require HR in embryos (Figure 5B).

### Modelling of a novel non-invasive control strategy based on *vasa*-driven HEG expression

We also modelled an alternative non-invasive vector control strategy, based on the observation that the Vas2GFP regulatory regions could drive expression in the earliest developmental stages of both male and female germlines. If a HEG was designed to target a germline-specific gene expressed in ovaries downstream of *vasa *which is required for female fertility, then *vasa *driven HEG expression in heterozygote females and cleavage of the target gene would lead to sterilization of those same females. In this approach the HEG would drive in the male germline without affecting male fitness and cause dominant sterility in heterozygote females (with penetrance depending upon the level of HEG activity). Unless homing rates are very low, maternal deposition has little effect in this strategy as heterozygote females are sterile, and the minimal contribution it provides to female sterilization becomes evident only in later generations. We modelled this approach and compared it to classical inundative strategies like SIT or RIDL (Figure 5C) [35-40]. In the simplest case of males and females having equal rates of cleavage and repair, such a HEG cannot invade a population from low frequencies. Measured in terms of the number of sterile females produced per male released, such a HEG-based approach can be several-fold more effective than Sterile Insect Technique (SIT) or Release of Insects carrying a Dominant Lethal (RIDL), depending upon the rates of cleavage and HR (Figure 5C).

## Discussion

Both sequence homology and analysis of the expression profile indicate that AGAP008578 is the *Anopheles gambiae *orthologue of the *Drosophila vasa *gene. Transgenic reporter strains utilizing different sequences upstream of the *vasa *gene provided information about the regulatory regions required to mirror the endogenous *vasa *transcription pattern. We have demonstrated that this region can be utilized to drive transgene expression in the mosquito germlines. Interestingly, we observed expression to be restricted to the late spermatogenic stages of the male germline with one of the two constructs generated (Vas1GFP) that only contains exon 1a of the 5'UTR and upstream sequences. The second construct, (Vas2GFP), that contains the entire 5'UTR and only 380 bp of upstream sequence directed eGFP germline expression throughout development including expression in the GSCs of both sexes. This indicates that regulatory elements essential for female germline expression are located within the 5' UTR of the *A. gambiae vasa *locus. Our data also suggest that elements important for expression in the early GSCs of both sexes map to this region. Regulatory elements important for female and early germline expression could function cooperatively or independently of each other. For example, expression in later stages of the female germline, may depend on expression in previous developmental stages although in males lack of expression in the GSCs of Vas1GFP testes does not abolish eGFP expression later on during spermatogenesis.

We have observed uniform eGFP distribution in ovarian nurse cells and in freshly laid embryos as a result of maternal deposition from transgenic Vas2GFP females. The localization pattern does not match the expected distribution of endogenous Vasa, as inferred from experiments in *Drosophila*. In this species Vasa protein localizes to the posterior end of the embryo, which will later develop into the pole plasm, whereas in the ovarian nurse cells it is found exclusively in the perinuclear region [27,29]. This localization to the posterior pole of the embryos requires a putative protein localisation motif of the Vasa N-terminus, which is absent from our eGFP reporter, but which has been shown to be essential for localization of a GFP fusion protein to the pole plasm of *Drosophila *embryos [30]. Such an amino acid sequence, if identified within the *Anopheles vasa *coding sequence could be used to specify transgene localization to the pole plasm although such localisation may interfere with transgene function.

In general, it is likely that strong expression in the female germline (especially in mature nurse cells of ovarian follicles) is tightly associated with embryonic maternal deposition. As gene drive systems may depend on regulatory elements that direct expression of effector molecules in female germ cells we modelled the effect of maternal deposition on the outcome of a HEG based strategy [12,34]. We found that fixation of HEGs that are active in both male and female germlines can occur in the target population even when HEGs are maternally deposited, regardless of whether this leads to homing or induces mutation of target genes in the embryo. However, the mean frequency of HEG alleles within the population at the time of fixation can be moderately reduced by maternal deposition if no homing occurs in embryos. If homing does occur in embryos our models suggest that the frequency of mean population fitness, i.e. the fraction of females in the population whose reproductive potential remains unaffected, reaches a lower level as opposed to strategies without maternal deposition. Previously described HEG-based driving constructs are predicted to invade a population even at low rates of homing [12], but with maternal deposition of homing endonucleases a minimum threshold of homing is required for such constructs to be invasive, i.e. be able to increase in frequency in successive generations. Apart from influencing HEG-based gene drive systems, the observation that Vas2GFP is able to deposit the products of transgenes into embryos may allow the construction of maternal-effect selfish genetic elements for *Anopheles *control [13].

Non-invasive control strategies like the Sterile Insect Technique (SIT) or Release of Insects carrying Dominant Lethal (RIDL) depend on the ability of released males to negatively affect the reproduction of the females they mate with. However, to achieve a substantial impact on the size of the target population these strategies rely on continuous large-scale releases because dominantly lethal genes or sterile males quickly disappear from the population. To substantially increase the efficiency of such strategies suggestions have been made to link the advantages of genetic drive with dominant sterility or lethality [35]. Here, we propose a mechanism for such a system: If a HEG was engineered to target a female fertility gene that exerts its function only in the female germline cells at a time point after initiation of transcription of the *vasa *promoter, expression of the HEG from the Vas2GFP regulatory regions would lead to dominant sterilization of females expressing the HEG. We used simple computational modelling to compare this approach to classic inundative control strategies. As the HEG will dominantly sterilize females but will spread in males it's persistence in the population is dramatically increased. Our models suggest that this approach remains non-invasive even at high homing rates and that the HEG will ultimately disappear. However, the effect on population reproduction, as measured by the amount of sterile females produced per released male, is predicted to be several fold higher than the effect of SIT or RIDL, depending on the rate of homing achieved. While the expression profile of the Vas2GFP regulatory region is well suited for the use in such a strategy, other early promoter elements may be equally utilized for this approach once available.

The Vas2GFP driven GFP transgene is an excellent germline marker for mosquitoes and allows expression in the germ line stem cells of both sexes. Expression in gonads can be observed throughout development from early larval stages to the reproductive tissues of adult mosquitoes. In addition to providing the opportunity of studying basic biological questions this expression pattern and the strong intensity of fluorescence make the Vas2GFP lines a suitable reporter system to identify compounds interfering with gonad development. Using a combination of high throughput fluorescent sorting of larvae and a combinatorial library it could therefore be used to screen for active substances e.g. inducing chemical sterilization. The injection of dsRNA species into embryos or adults would also allow studying other genetic aspects of gonad development.

## Conclusion

In recent years, several genetic control strategies have been proposed as methods for reducing the transmission of mosquito-borne diseases. These often rely on the ability of the control trait to increase in frequency once released, a feature that is inherently determined by its function in the mosquito germlines. Our results show that the regulatory regions of the *An. gambiae vasa *gene are suitable for driving transgenes in the early germlines of both sexes and for depositing their products in embryos of transgenic females. We developed computational models to assess whether an invasive HEG-based gene drive system can function in the presence of maternal deposition when driven by the *vasa *regulatory elements and describe a novel non-invasive control strategy that couples female sterilisation with transmission ratio distortion through males.

## Methods

### Plasmid Construction

For Vas1GFP the 1957 bp used as putative promoter and 5'UTR was amplified from G3 genomic DNA using primers Vas5'fwd1ageI (AGACCGGTGCGCACATTACCTGCGATCACG) and Vas5'rev1ncoI (GTTCCCATGGCTGAGTTTTGAAGTACTATTC). The resulting fragment was cloned into pEGFP (Clontech) using *Agel *and *Ncol*, 5' of the eGFP coding region. The Vas3'UTR and 3'flanking sequences (1000 bp) were amplified with primers Vas3'fwd2notI (GTGCGGCCGCCTTGGGGTGGGGTTGTTATGTGTTG) and Vas3'rev1ecorI (CAGAATTCGAAAATGTGGCCATTAACAGCAG) and inserted 3' of the eGFP stop codon using *Not*I and *Eco*RI. The 3.6 kb cassette was removed from the resulting plasmid with *Agel *and *Eco*Rl and subcloned in the shuttle vector pslfa11180fa. Finally, the Vas1GFP cassette was cloned into pBac3xP3RFP with *Asc*I. For Vas2GFP the putative promoter and entire *vasa *5'UTR (2291 bp) were amplified using primers Vas5'fwd4ageIascI (GGACCGGTGGCGCGCCATGTAGAACGCGAGCAAATTCTTTTCC) and Vas5'revcdspciI (CCGCACATGTTTCCTTTCTTTATTCACC). The fragment was digested with *Agel *and *Pci*l and cloned into pEGFP digested with *Agel *and *Ncol*. The 3'UTR and 3'flanking sequence (1000 bp), amplified with Vas3'fwd2notI and Vas3'rev5ascInotI GGCGGCCGCGGCGCGCCGAAAATGTGGCCATTAACAGCAG), was inserted in the above construct with *Not*l and finally the entire cassette was removed with *Asc*I and cloned into pBac3xP3RFP. The resulting constructs pBac{3xP3RFP}Vas1GFP and pBac{3xP3RFP}Vas2GFP were called Vas1GFP and Vas2GFP respectively.

### Development of Transgenic lines

Transgenic lines were developed as described [21,41]. *A. gambiae *embryos (strain G3) were injected using a Femtojet Express injector and sterile Femtotips (Eppendorf) with a mixture of 0.2 μg/μl of plasmid and 0.8 μg/μl of piggyback helper RNA. The hatched larval survivors were screened for transient expression of the 3xP3 RFP marker and only transients were grown up and crossed to wild type mosquitoes. The progeny of these crosses were analyzed for RFP fluorescence and independent lines were established. Chromosomal integration sites were established by inverse PCR (see Additional File 3).

### Transcriptional profiling

Total RNA was prepared from dissected mosquito tissues using TRI reagent (Ambion). Following RQ1 DNAse (Promega) treatment, cDNA was prepared using Superscript II (Invitrogen) and oligodT primers (Invitogen) following the manufacturer's instructions. 0.5 μl of the resulting cDNA samples was used for PCR using Phusion DNA Polymerase (Finnzymes). PCR primers: S7: F-GGCGATCATCATCTACGTGC, R-GTAGCTGCTGCAAACTTCGG; beta2-tubulin: F-GCCCGTAGGAATCGCACGTATTCGG, R-GATCGAGAACGTGTTCATGATGCGGTC; nanos: F-GTTTGCAAGCCGTGCAGCAAAGGG, R-CCCTTACGGTGTATCTTATGCCGCCG; vasa: F-CCAGCCGAATAGTACTTCAAAACTCAGC, R-CGCAACACATAACAACCCCACCCC; eGFP: F-GAGCAAGGGCGAGGAGCTGTTCACC, R-CTTGTACAGCTCGTCCATGCCGAGAG.

### Microscopy

Dissected gonads were fixed in methanol-free 4% formaldehyde (Pierce) in PBS for 30 min and washed 3 times for 15 min in 0.1% Tween-20 PBS. Gonads were then mounted on fresh slides containing Vectashield mounting medium with DAPI (Vectorlabs. Inc.) with cover slips. Multiplane z-stacks were collected by confocal analysis (SP5; Leica). Embryos deriving from crosses of Vas2GFP heterozygote mothers or fathers crossed respectively to wild type were subjected to fluorescent analysis, once exochorions were removed essentially as described [42].

### Maternal Deposition

To test for maternal deposition we crossed virgin heterozygote transgenic Vas2GFP females with wild type males and as a control, heterozygote transgenic Vas2GFP males with virgin wild type females. Mosquitoes were mated for 5 days and females were allowed to lay eggs 3 days after a blood meal. mRNA was extracted from approximately 100 eggs at several time points post-oviposition and subjected to RT-PCR analysis using GFP, *vasa *and S7 primer pairs. For fluorescent scoring hatchlings resulting from the above crosses were scored for GFP and RFP fluorescence immediately after hatching and after 5 days in the 2^nd ^instar stage.

### Computational modelling

We modelled a randomly mating population in which we assumed three classes of alleles at a female fertility locus: wild type (*wt*), HEG-containing (*HEG*) and the misrepaired (*M*). The *M *allele is nonfunctional and not cleavable by the endonuclease. In the model, *M *alleles are not functional based on the assumption that the HEG recognition sequence is located in a highly conserved region of the target gene. *HEG/HEG*, *HEG/M*, and *M/M *females sterile, but males are unaffected by loss of gene function. In the germlines of male and female *HEG/wt *mosquitoes the *wt *allele is cleaved with probability *c*, and then converted into a *HEG *allele with probability *h*_*g*_, or into an *M *allele with probability 1 – *h*_*g*_, where *h *is the rate of homing (*h*_*g *_denotes activity in the germline). Use of the homologous chromosome to convert the *wt *allele into a *HEG *allele is driven by homologous repair (HR). *M *alleles can be generated *de novo *on cleaved *wt *alleles by NHR (e.g. Non-Homologous End Joing, NHEJ) or inaccurate HR. *wt *alleles can also be converted into *M *alleles by accurate HR, if an *M *allele is available as repair template – a situation that can only occur in embryos, where the endonuclease protein is provided through maternal deposition. In all cases, the variable rates of the model, *c h*_*g*_, *h*_*e *_and *D*, relate only to the probability with which a *wt *allele is converted into either a *HEG *or an *M *allele, and not to the particular pathway that achieves the conversion – HR or NHR which were used for simplicity. The two computational models generated differ in the class of female fertility gene targeted: (i) those that are only needed in somatic tissues, so that cleavage and repair in the germline of heterozygous females has no effect on their fertility or viability; and (ii) those that are required in the female germline after HEG expression, so that cleavage and repair in the germline of heterozygous females will sterilize those same females. Inherited *wt *alleles of embryos arising from *HEG/wt *mothers can be further cleaved by deposited homing endonucleases, with probability *D *and then converted into *HEG *alleles with probability *h*_*e *_or *M *alleles with probability 1 – *h*_*e *_(*h*_*e *_denotes activity in the embryo). As there is no data about the consequences of homing endonuclease maternal deposition in embryos we have modelled possible two scenarios: (i) cut sites are repaired with rates identical to those assumed for the germline (*h*_*e *_= *h*_*g*_), or (ii) where all sites are repaired by non homologous repair (*h*_*e *_= 0). The algorithms and model data were generated in the Mathematica software suite (Wolfram Research) and are presented in more detail in Additional file 4.

## Abbreviations

HEG: homing endonuclease gene; UTR: untranslated region; DSB: double-stranded break; HR: homologous repair; NHR: non-homologous repair; GSC: germline stem cell; SSC: somatic stem cell; SIT: sterile insect technique; RIDL: release of insects carrying dominant lethal; RT-PCR: reverse transcription polymerase chain reaction.

## Authors' contributions

PAP and NW conceived and designed the experiments. PAP, NW and MM performed the experiments. PAP and NW generated the transgenic lines. PAP and NW analyzed the data. PAP and AB generated the computational models. PAP, NW and AC wrote the paper. PAP initiated the project. AC supervised and inspired the project. All authors read and approved the final manuscript.

## Supplementary Material

Additional file 1 **ClustalW alignment of Drosophila melanogaster Vasa with its predicted Anopheles gambiae orthologue AGAP008578**. Highlighted under the alignment are the known Drosophila motifs. The two vasaspecific RNA interacting residues of Drosophila (Arg403 and Glu497) and their conserved orthologue residues in AGAP008578 (Arg334 and Glu493) are highlighted in red. Click here for file

Additional file 2 **eGFP expression analysis in transgenic Vas1GFP and Vas2GFP mosquitoes**. A) Expression pattern of eGFP from the Vas1GFP and Vas2GFP reported constructs in male and female larvae (L4 larval stage). Male and female Vas1GFP larvae showed two distinct eGFP expression phenotypes. The testes of developing male larvae were clearly expressing eGFP (top left panel), whilst female larvae did not show eGFP fluorescence (top right panel). Male and female Vas2GFP larvae showed an identical eGFP expression pattern until late larval stages, when testes of male larvae adopted a typical sherical shape (bottom left panel), whilst female ovaries adopted GFP fluorescing longitudinal structures (bottom right panel). The microphotographs also show superimposed to the eGFP signal the RFP signal generated by the 3XP3-RFP transformation marker. B) Transmission (TM) and fluorescence (GFP) microphotographs showing eGFP fluorescence in Vas1GFP (left panel), Vas2GFP (middle panel) and wild type (WT – right panel) mature spermatozoa examined in ruptured spermathecae disected from WT females mated with either Vas1GFP, Vas2GFP or wild type males. C) Relationship between eGFP fluorescence phenotype and adult sex in Vas1GFP mosquitoes. D) Detection of eGFP protein in transgenic Vas1GFP tissues. The expression profile of Vas1GFP in transgenic mosquito adults was confirmed by western blotting. Equal numbers (five) of Vas1GFP male and females mosquitoes from Line 1 were dissected to generate tissue lisates from testes pairs (T), male gonad-less carcasses (MC), female gonad-less carcasses (FC), ovary pairs from non-blood fed female mosquitoes(NBF), ovary pairs 24 hours post-blood feeding (24 hr) and ovary pairs 48 hours post-blood feeding (48 hr). To test the sensitivity of this assay in detecting eGFP protein we also overloaded one lane with 10 complete males in which the testes had not been removed (M). The lysates were run on SDS page blotted and tested against with either anti-eGFP or anti-α-tubulin antibodies. Click here for file

Additional file 3 **Chromosomal integration sites of transgenic lines**. Inverse PCR was performed on Vas1GFP and Vas2GFP transgenic lines using standard protocols. Click here for file

Additional file 4 **Additional Methods**. The additional document covers supplementary methods on western blotting and presents the computational models in more detail. Click here for file
