# Supplementary figures and images for: The vasa regulatory region mediates germline expression and maternal transmission of proteins in the malaria mosquito Anopheles gambiae: a versatile tool for genetic control strategies
Source: BMC Mol Biol. 2009 Jul 2;10:65. doi: 10.1186/1471-2199-10-65 (PMC2713240; doi:10.1186/1471-2199-10-65)

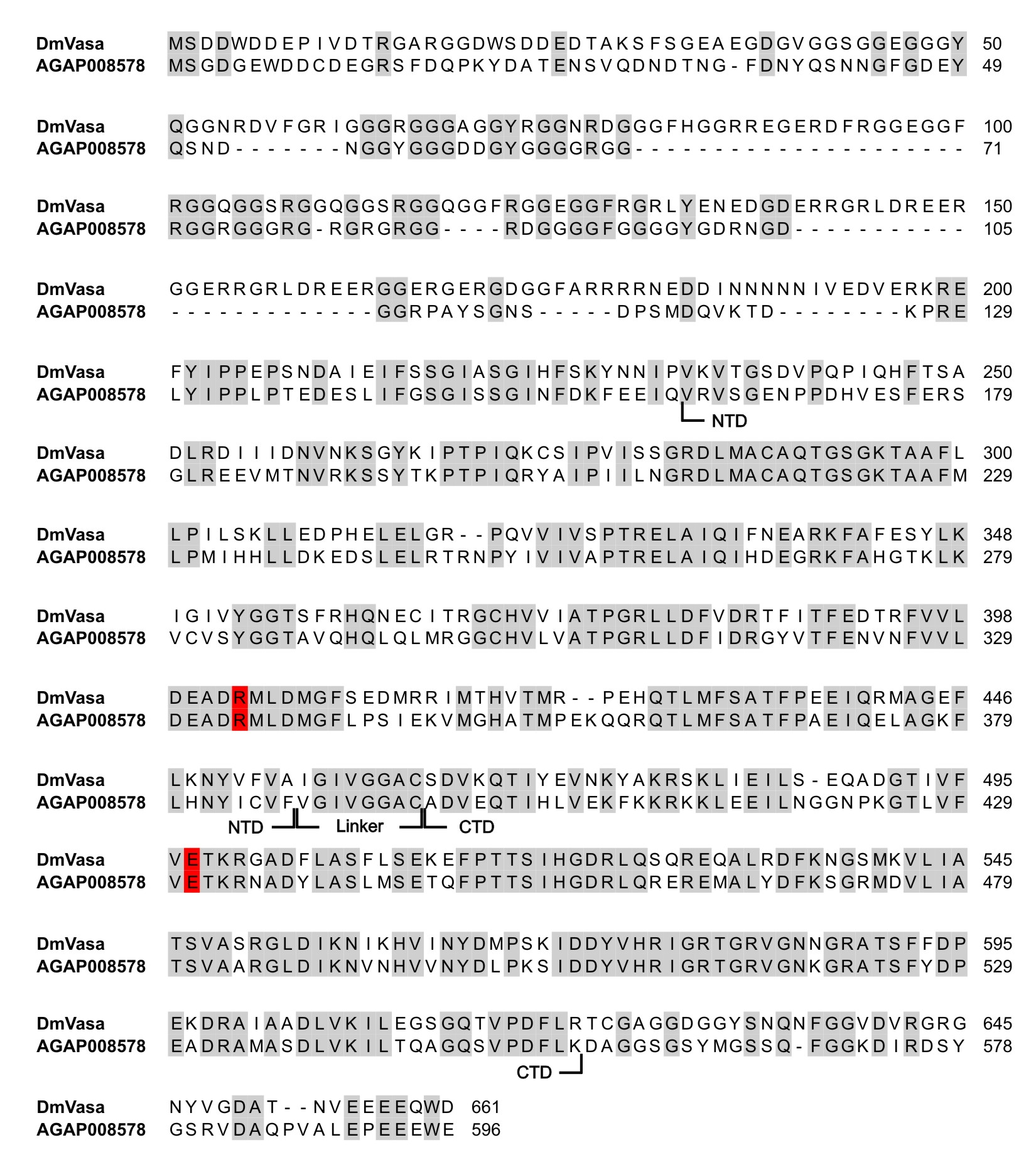

Supplement: Additional file 1 — ClustalW alignment of Drosophila melanogaster Vasa with its predicted Anopheles gambiae orthologue AGAP008578. Highlighted under the alignment are the known Drosophila motifs. The two vasaspecific RNA interacting residues of Drosophila (Arg403 and Glu497) and their conserved orthologue residues in AGAP008578 (Arg334 and Glu493) are highlighted in red. [file 1471-2199-10-65-S1.jpeg]

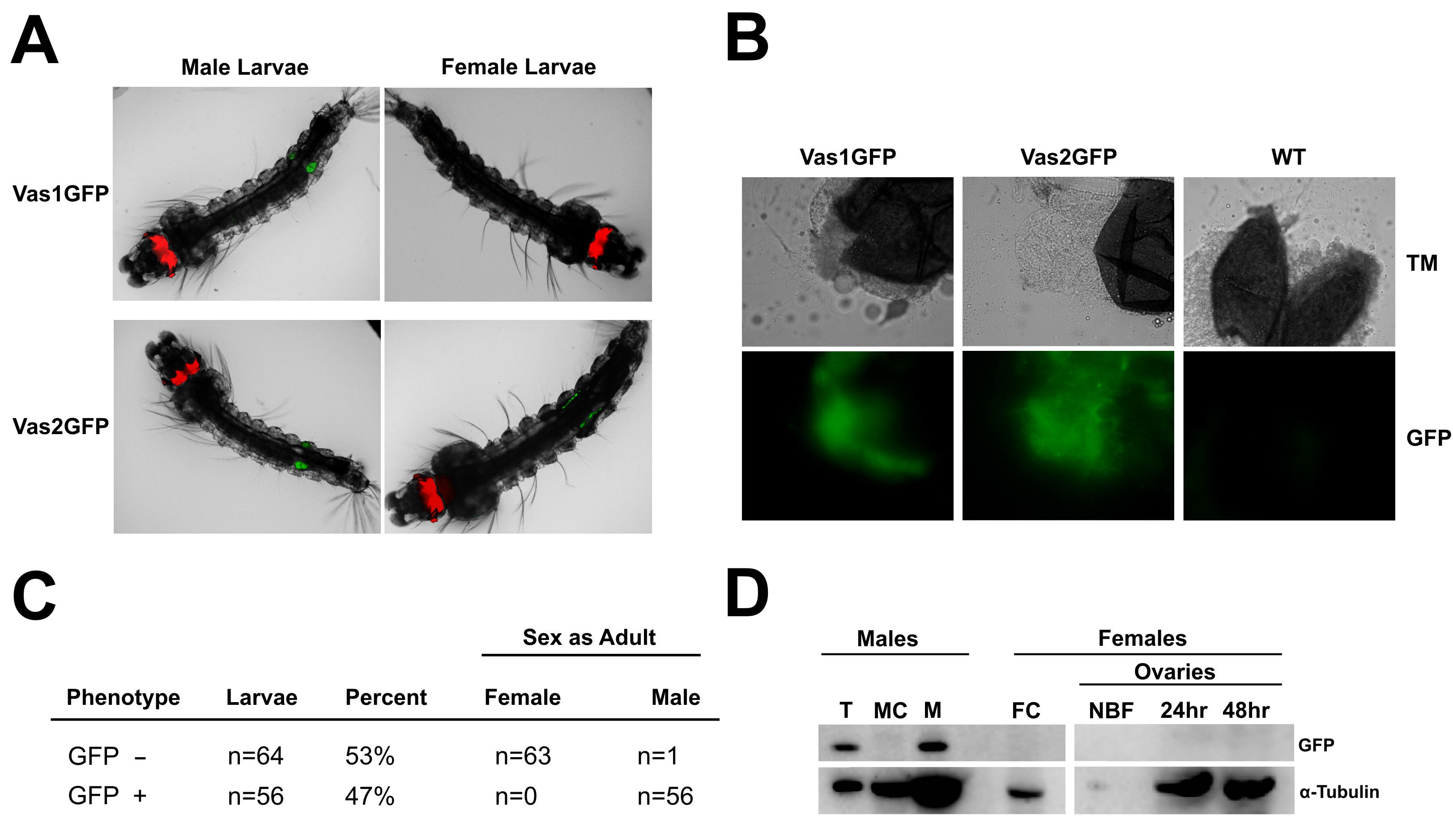

Supplement: Additional file 2 — eGFP expression analysis in transgenic Vas1GFP and Vas2GFP mosquitoes. A) Expression pattern of eGFP from the Vas1GFP and Vas2GFP reported constructs in male and female larvae (L4 larval stage). Male and female Vas1GFP larvae showed two distinct eGFP expression phenotypes. The testes of developing male larvae were clearly expressing eGFP (top left panel), whilst female larvae did not show eGFP fluorescence (top right panel). Male and female Vas2GFP larvae showed an identical eGFP expression pattern until late larval stages, when testes of male larvae adopted a typical sherical shape (bottom left panel), whilst female ovaries adopted GFP fluorescing longitudinal structures (bottom right panel). The microphotographs also show superimposed to the eGFP signal the RFP signal generated by the 3XP3-RFP transformation marker. B) Transmission (TM) and fluorescence (GFP) microphotographs showing eGFP fluorescence in Vas1GFP (left panel), Vas2GFP (middle panel) and wild type (WT – right panel) mature spermatozoa examined in ruptured spermathecae disected from WT females mated with either Vas1GFP, Vas2GFP or wild type males. C) Relationship between eGFP fluorescence phenotype and adult sex in Vas1GFP mosquitoes. D) Detection of eGFP protein in transgenic Vas1GFP tissues. The expression profile of Vas1GFP in transgenic mosquito adults was confirmed by western blotting. Equal numbers (five) of Vas1GFP male and females mosquitoes from Line 1 were dissected to generate tissue lisates from testes pairs (T), male gonad-less carcasses (MC), female gonad-less carcasses (FC), ovary pairs from non-blood fed female mosquitoes(NBF), ovary pairs 24 hours post-blood feeding (24 hr) and ovary pairs 48 hours post-blood feeding (48 hr). To test the sensitivity of this assay in detecting eGFP protein we also overloaded one lane with 10 complete males in which the testes had not been removed (M). The lysates were run on SDS page blotted and tested against with either anti-eGFP or anti-α-tubulin a [file 1471-2199-10-65-S2.jpeg]

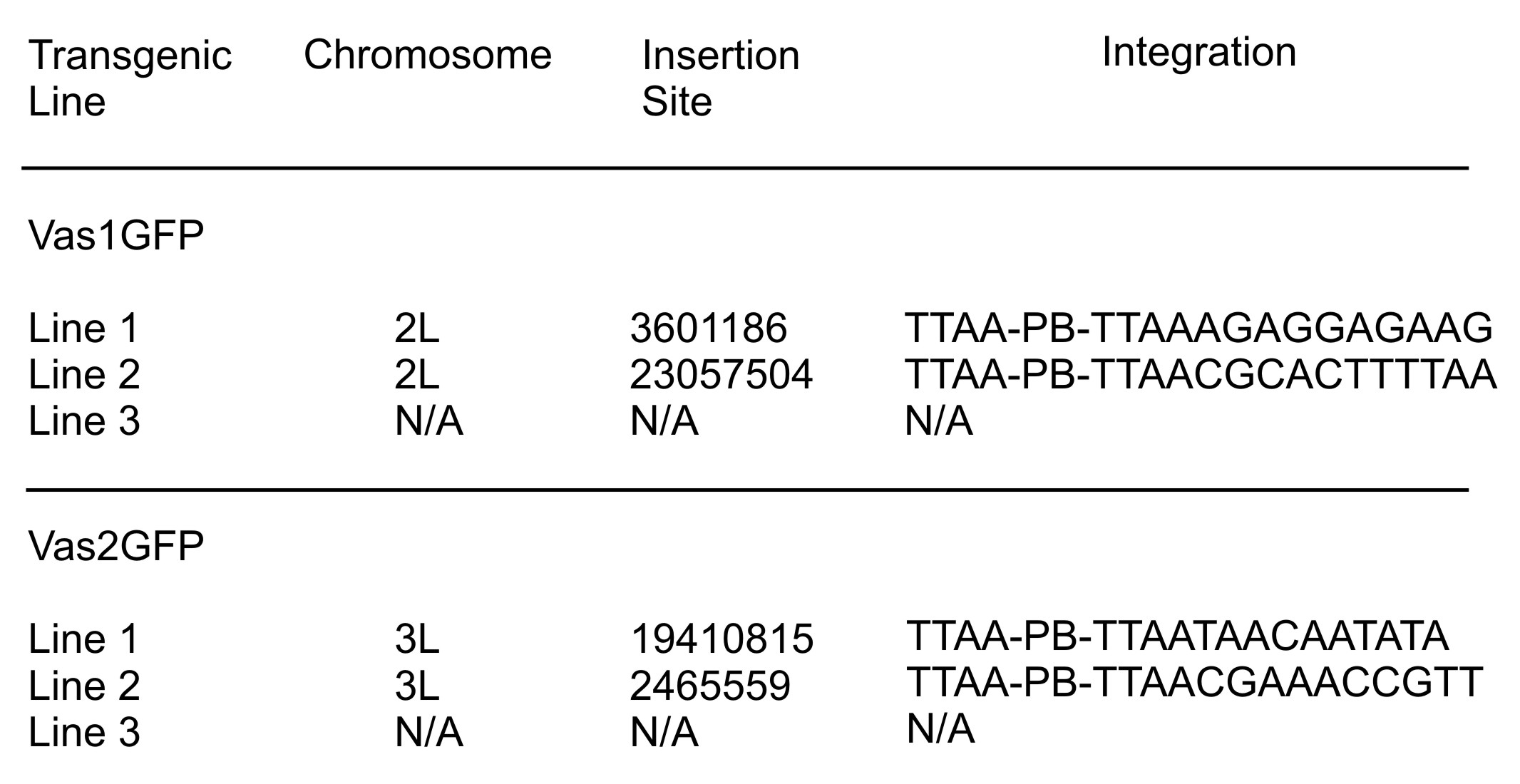

Supplement: Additional file 3 — Chromosomal integration sites of transgenic lines. Inverse PCR was performed on Vas1GFP and Vas2GFP transgenic lines using standard protocols. [file 1471-2199-10-65-S3.jpeg]
