# Supplementary material for: The vasa regulatory region mediates germline expression and maternal transmission of proteins in the malaria mosquito Anopheles gambiae: a versatile tool for genetic control strategies
Source: BMC Mol Biol. 2009 Jul 2;10:65. doi: 10.1186/1471-2199-10-65 (PMC2713240; doi:10.1186/1471-2199-10-65)
Supplement: Additional file 4 — Additional Methods. The additional document covers supplementary methods on western blotting and presents the computational models in more detail. [file 1471-2199-10-65-S4.pdf]

ADDITIONAL METHODS

Western Blotting:

Male and female transgenic Vas1GFP mosquitoes were dissected in PBS to separate the ovaries or testes from the remaining gonad-less carcasses and placed in PBS with EDTA-free Protease Inhibitor Cocktail (Roche Applied Science). For female ovaries a time series containing lysates of non-bloodfed and 24 or 48 hours post-bloodfeeding was also collected. Samples were separated on 10–12% SDS-PAGE gels followed by electro-transfer to nitrocellulose membranes and incubated with antibodies against eGFP (1:3000 mouse monoclonal anti-GFP Living Colours JL-8) and as a loading control against alpha-tubulin (1:1000 mouse monoclonal anti-α-Tubulin Sigma Aldrich DMIA). Primary antibodies were detected using anti-mouse IgG HRP-conjugated secondary antibody and visualized by Western Lightning Chemiluminescence Reagent Plus kit (Perkin Elmer). Blots were stripped in 62.5 mM Tris-HCl, pH 6.7, 2% SDS, and 100 mM Mercaptoethanol at 60°C for 1 h.

Computational Modelling:

Targeting a somatic female fertility gene (invasive approach)

At the target locus there are 3 different alleles: wildtype (*wt*), HEG-containing knockout (*HEG*), and misrepaired mutant knockout (*M*). Therefore there are 6 genotypes: *HEG*/*HEG* (genotype1), *HEG*/*wt* (genotype2), *wt*/*wt* (genotype3), *M*/*HEG* (genotype4), *M*/*wt* (genotype5), *M*/*M* (genotype6).

Let the frequencies of these 6 genotypes in adult males be m1, m2, m3, m4, m5, m6, and in fertile adult females be f1, f2, f3, f4, f5, f6 (we exclude the sterile females because they do not contribute to the next generation). Then the frequencies of the 6 genotypes in the embryos of the next generation (after maternal deposition has had its effect) will be:

$\mathrm{e1}' = \frac{1}{4}\mathrm{f2}\left(2\left(1 + c(-1 + c)Dh_e + h_g\right)\right)\mathrm{m1} + \mathrm{m2} + \mathrm{m4} + c\left(h_g\left(\left(2 + ch_g\right)\mathrm{m2} + \mathrm{m4}\right) + Dh_e\left(-2(-1 + c)\left(1 + ch_g\right)\mathrm{m2} + 2\left(\mathrm{m3} + ch_g\mathrm{m3}\right) + \mathrm{m4} - c\mathrm{m4} + \mathrm{m5} + ch_g\mathrm{m5}\right)\right)$

$\mathrm{e2}' = \frac{1}{4}((2\mathrm{f3} + \mathrm{f5})(2\mathrm{m1} + \mathrm{m2} + ch_g\mathrm{m2} + \mathrm{m4}) + \mathrm{f2}(-1 + cD(1 + h_e))(2(-1 + c)\mathrm{m1} + 2(-1 + c)(1 + ch_g)\mathrm{m2} - 2\mathrm{m3} - \mathrm{m4} - \mathrm{m5} + c(\mathrm{m4} - h_g(2\mathrm{m3} + \mathrm{m5}))))$

$\mathrm{e3}' = \frac{1}{4}(-2\mathrm{f3} - \mathrm{f5} + (-1 + c)\mathrm{f2}(-1 + cD(1 + h_e))^2)((-1 + c)\mathrm{m2} - 2\mathrm{m3} - \mathrm{m5})$

$\mathrm{e4}' = \frac{1}{4}(-2c^3D\mathrm{f2}h_g\mathrm{m2} + \mathrm{f5}(2\mathrm{m1} + \mathrm{m2} + \mathrm{m4}) + c^2\mathrm{f2}(-2(-1 + h_g)h_g\mathrm{m2} + D(-2\mathrm{m1} + 2(-1 + h_g)\mathrm{m2} + 2h_g\mathrm{m3} - \mathrm{m4} + h_g\mathrm{m5})) + \mathrm{f2}(\mathrm{m4} + \mathrm{m5} + 2\mathrm{m6}) + c(\mathrm{f5}h_g\mathrm{m2} + \mathrm{f2}(2(1 + D - h_g)\mathrm{m1} + 2\mathrm{m2} + \mathrm{m4} + D(2\mathrm{m2} + 2\mathrm{m3} + \mathrm{m4} + \mathrm{m5}) + h_g(-2\mathrm{m2} + \mathrm{m5} + 2\mathrm{m6}))))$

$\mathrm{e5}' = \frac{1}{4}\left(-2c^4D^2\mathrm{f2}(1 + h_e)^2\mathrm{m2} + 2c^3D\mathrm{f2}(1 + h_e)\left(\left(2 + 2D(1 + h_e) - h_g\right)\mathrm{m2} + D(1 + \mathrm{he})(2\mathrm{m3} + \mathrm{m5})\right) + c^2\mathrm{f2}\left(-2\left(1 + D(1 + h_e)\left(3 + D + Dh_e - h_g\right) - h_g\right)\mathrm{m2} - D(1 + h_e)\left(2\left(3 + 2D(1 + h_e) - h_g\right)\mathrm{m3} - \mathrm{m4} + \left(2 + 2D(1 + h_e) - h_g\right)\mathrm{m5} - 2\mathrm{m6}\right)\right) + c\left(-\left(2\mathrm{f3}(-1 + \mathrm{hg}) + \mathrm{f5}h_g\right)\mathrm{m2} + \mathrm{f2}\left(2\left(1 + D + Dh_e - h_g\right)\mathrm{m2} + 2\mathrm{m3} - \mathrm{m4} - h_g(2\mathrm{m3} + \mathrm{m5}) + D(1 + h_e)(4\mathrm{m3} - \mathrm{m4} + \mathrm{m5} - 2\mathrm{m6}) - 2\mathrm{m6}\right)\right) + (\mathrm{f2} + 2\mathrm{f3})(\mathrm{m4} + \mathrm{m5} + 2\mathrm{m6}) + \mathrm{f5}\left(\mathrm{m2} + 2\mathrm{m3} + \mathrm{m4} + 2(\mathrm{m5} + \mathrm{m6})\right)\right)$

$\mathrm{e6}' = \frac{1}{4}(c^4D^2\mathrm{f2}(1 + h_e)^2\mathrm{m2} - c^3D\mathrm{f2}(1 + h_e)(2(1 + D + Dh_e - h_g)\mathrm{m2} + D(1 + h_e)(2\mathrm{m3} + \mathrm{m5})) + c^2\mathrm{f2}((1 + D + Dh_e - h_g)^2\mathrm{m2} + D(1 + h_e)(2(1 + D + Dh_e - h_g)\mathrm{m3} - \mathrm{m4} + (D + Dh_e - h_g)\mathrm{m5} - 2\mathrm{m6})) + \mathrm{f5}(\mathrm{m4} + \mathrm{m5} + 2\mathrm{m6}) + c(-\mathrm{f5}(-1 + h_g)\mathrm{m2} + \mathrm{f2}(1 + D + Dh_e - h_g)(\mathrm{m4} + \mathrm{m5} + 2\mathrm{m6})))$

where *c* is the rate of cleavage; cleaved alleles in the germline are repaired by homologous repair (HR) with probability h_g or by non-homologous repair (NHR) with probability 1-h_g; cleaved alleles in the embryo are repaired by HR with probability h_e or by NHR with probability 1- h_e; and *D* is the probability of maternal deposition (0 or 1 in the simulations presented in the text).

All males are fertile, but three of the female genotypes are sterile (*HEG*/*HEG*, *HEG*/*M*, and *M*/*M*). Genotype frequencies among fertile females are therefore:

$$\mathrm{f1}' = 0$$

$$\mathrm{f2}' = \frac{\mathrm{e2}'}{\mathrm{e2}'+\mathrm{e3}'+\mathrm{e5}'}$$

$$\mathrm{f3}' = \frac{\mathrm{e3}'}{\mathrm{e2}'+\mathrm{e3}'+\mathrm{e5}'}$$

$$\mathrm{f4}' = 0$$

$$\mathrm{f5}' = \frac{\mathrm{e5}'}{\mathrm{e2}'+\mathrm{e3}'+\mathrm{e5}'}$$

$$\mathrm{f6}' = 0$$

The frequencies of the *HEG*, *M* and *wt* alleles (at the embryo stage) are then:

$$\mathrm{e1} + \frac{\mathrm{e2}}{2} + \frac{\mathrm{e4}}{2}$$

$$\frac{\mathrm{e4}}{2} + \frac{\mathrm{e5}}{2} + \mathrm{e6}$$

$$\frac{\mathrm{e2}}{2} + \mathrm{e3} + \frac{\mathrm{e5}}{2}$$

respectively, and the frequency of sterile females is:

$$\mathrm{S} = \mathrm{e1} + \mathrm{e4} + \mathrm{e6}$$

To determine the criteria for invasion we derived an expression for p[3]/p[2], where p[i] is the frequency of the HEG in the ith generation, and took the limit where the introduction frequency was vanishingly small. When there is homing in the embryos (he = hg), the line of neutrality for the HEG is given by

$$\mathrm{hg} = \frac{5 - 3c - \sqrt{25 - 22c + 5c^2}}{2(-2c + c^2)}$$

whereas when there is no homing in the embryo (he = 0), the line of neutrality is given by

$$\mathrm{hg} = -\frac{1}{2(-2 + c)}$$

The population mean fitness presented in the text was calculated as the proportion of females that are fertile, which is:

$$\mathrm{e2} + \mathrm{e3} + \mathrm{e5}$$

Targeting a germline female fertility gene (non-invasive approach)

If the target locus is a germline fertility gene then *HEG*/*wt* females can be sterilized by the action of the HEG in the germline. In this case the frequencies of the 6 genotypes among embryos will be:

e1′ = $\frac{1}{4}\mathrm{f2}(2(\mathrm{m1} + cDh_e\mathrm{m1}) + \mathrm{m2} + \mathrm{m4} + c(h_g\mathrm{m2} + Dh_e((2 + c(-1 + h_g))\mathrm{m2} + 2\mathrm{m3} + \mathrm{m4} + \mathrm{m5})))$

e2′ = $\frac{1}{4}((2\mathrm{f3} + \mathrm{f5})(2\mathrm{m1} + \mathrm{m2} + ch_g\mathrm{m2} + \mathrm{m4}) - \mathrm{f2}(-1 + cD(1 + h_e))(2\mathrm{m1} + (2 + c(-1 + h_g))\mathrm{m2} + 2\mathrm{m3} + \mathrm{m4} + \mathrm{m5}))$

e3′ = $-\frac{1}{4}\left(2\mathrm{f3} + \mathrm{f5} + \mathrm{f2}\left(-1 + cD(1 + h_e)\right)^2\right)\left((-1 + c)\mathrm{m2} - 2\mathrm{m3} - \mathrm{m5}\right)$

e4′ = $\frac{1}{4}(c^2D\mathrm{f2}(-1 + h_g)\mathrm{m2} + \mathrm{f5}(2\mathrm{m1} + \mathrm{m2} + \mathrm{m4}) + c((\mathrm{f2} - \mathrm{f2}h_g + \mathrm{f5}h_g)\mathrm{m2} + D\mathrm{f2}(2\mathrm{m1} + 2\mathrm{m2} + 2\mathrm{m3} + \mathrm{m4} + \mathrm{m5})) + \mathrm{f2}(\mathrm{m4} + \mathrm{m5} + 2\mathrm{m6}))$

e5′ = $\frac{1}{4}(2c^3D^2\mathrm{f2}(1 + h_e)^2\mathrm{m2} - c^2D\mathrm{f2}(1 + h_e)((3 + 2D(1 + h_e) - h_g)\mathrm{m2} + 2D(1 + h_e)(2\mathrm{m3} + \mathrm{m5})) + c(-(2\mathrm{f3}(-1 + h_g) + \mathrm{f5}h_g)\mathrm{m2} + \mathrm{f2}((1 + 2D(1 + h_e) - h_g)\mathrm{m2} + D(1 + h_e)(4\mathrm{m3} - \mathrm{m4} + \mathrm{m5} - 2\mathrm{m6}))) + (\mathrm{f2} + 2\mathrm{f3})(\mathrm{m4} + \mathrm{m5} + 2\mathrm{m6}) + \mathrm{f5}(\mathrm{m2} + 2\mathrm{m3} + \mathrm{m4} + 2(\mathrm{m5} + \mathrm{m6})))$

e6′ = $\frac{1}{4}(-c^3D^2\mathrm{f2}(1 + h_e)^2\mathrm{m2} + c^2D\mathrm{f2}(1 + h_e)((1 + D + Dh_e - h_g)\mathrm{m2} + D(1 + h_e)(2\mathrm{m3} + \mathrm{m5})) + \mathrm{f5}(\mathrm{m4} + \mathrm{m5} + 2\mathrm{m6}) + c(-\mathrm{f5}(-1 + h_g)\mathrm{m2} + D\mathrm{f2}(1 + h_e)(\mathrm{m4} + \mathrm{m5} + 2\mathrm{m6})))$,

where all symbols have the same definition as above.

Again, the genotype frequencies of the adults are the same as for the embryos. All males are fertile, but three of the female genotypes are sterile (*HEG*/*HEG*, *HEG*/*M*, and *M*/*M*). Genotype frequencies among the subpopulation of females that are fertile are:

$$f1' = 0$$

$$f2' = \frac{e2' - ce2'}{e2' - ce2' + e3' + e5'}$$

$$f3' = \frac{e3'}{e2' - ce2' + e3' + e5'}$$

$$f4' = 0$$

$$f5' = \frac{e5}{e2' - ce2' + e3' + e5'}$$

$$f6' = 0$$

The ce2 term in the equation for f2' arises because *HEG*/*wt* females can be sterilised as a result of HEG-induced cleavage and knockout mutation of the target gene in the germline.

The frequencies of the *HEG*, *M* and *wt* alleles (at the embryo stage) are then:

$$\mathrm{e1} + \frac{\mathrm{e2}}{2} + \frac{\mathrm{e4}}{2}$$

$$\frac{\mathrm{e4}}{2} + \frac{\mathrm{e5}}{2} + \mathrm{e6}$$

$$\frac{\mathrm{e2}}{2} + \mathrm{e3} + \frac{\mathrm{e5}}{2}$$

respectively, and the frequency of sterile females is:

$$\mathrm{S} = \mathrm{e1} + c\mathrm{e2} + \mathrm{e4} + \mathrm{e6}$$

To calculate the total number of sterile females produced per released male in the *t* generations after release, we simulated a single introduction of homozygous HEG/HEG males and calculated

$$\frac{\sum_{i=1}^{t} S[i]}{\mathrm{init}}$$

where S[*i*] is the frequency of sterile females in the ith generation after release and *init* is the initial frequency of the released males, here taken to be 0.0001.
